# 

**Published:** 1891-07-18

**Authors:** 


### NOTICE TO CORRESPONDENTS.

All MS., Letters, Books for Review, and other matters intended for the Editor should be addressed The Editor, The Lodge, Porchester Square, London, W.

The Editor cannot undertake to return rejected M.S., even when accompanied by stamped directed envelope.

**Notice to Advertisers and Subscribers.**

All Advertisements, Orders for Copies of The Hospital, and Business Communications should be addressed to The Publisher, *not to the Editor*, The Hospital, 140, Strand, W.C.

The Cost of Subscription, post free, is as follows:—Per week, 1½d.; per quarter, 1s. 8d.; per half-year, 3s. 3d.; per annum, 6s. 6d. Foreign:—Per annum, 8s. 8d.; payable, in all cases, in advance.

**NOTICE.—The Index for Vol. IX., (ending March, 1891) is on sale at the Office of this Journal, price 2½d., post free.**

### General Charities.

[This Column is devoted to an account of work of charitable institutions other than Medical Charities. The Editor will be glad to receive reports or news of work at 140, Strand. *Philanthropy* should be plainly written on the envelope.]

The Goldsmiths' Company have sent £100 to the Hon. Alfred Lyttelton, Treasurer of **THE CHILDREN'S COUNTRY HOLIDAYS FUND.**

The Annual Fete of the Homes for Little Boys Farningham, which was announced to take place on the 18th inst., will not be held; in its place there will be a festival at Swanley later in the season, the date o which has not yet been fixed, in order to inaugurate the Morrison Home, which is approaching completion. The Summer Fête of the Earlswood Asylum is announced for Thursday, the 23rd inst.

The Prince of Wales, accompanied by the Princess, will lay the foundation stone of the **POLYTECHNIC INSTITUTE FOR SOUT H-WEST LONDON**, on Thursday, the 23rd inst., at 3.30 p.m. The Committee appeal urgently to the inhabitants of the South-west district, in view of the approaching ceremony, to aid in reducing the sum still required to meet the £50,000 promised by the Charity Commissioners, which amounts to more than £17,000. The conflicting claims of so many institutions in London will not make it easy, we fear, for the committee to accomplish their desire to be able to announce a large reduction in this deficit on the day of the ceremony. The offices are at 47, Duke Street, St. James's, S.W.

People have during recent years fixed their attention so closely upon the needs of the East-end of London that the majority seem to have forgotten that the West-end has its spots which are not altogether paradises of happiness. It is all the more creditable, therefore, to Miss Meredith Brown that she should interest herself actively in philanthropic effort amongst the congested and poor populations of such parts as Lisson Grove. The **SHAFTESBURY INSTITUTE AND LABOUR HOME FOR LADS**, 21, Bell Street, Edgware Road, which has been started under her auspices, is a kindred institution to that established in 1888 for working girls in Harrow Street. It is intended only to assist those boys who have an earnest desire to do better, and a record is kept of their previous history and future conduct. For every bundle of wood they chop and tie they receive one penny. Money is still needed to complete the purchase of the Home and to carry on the work.

The latest report of the **NATIONAL REFUGES FOR HOMELESS AND DESTITUTE CHILDREN** shows that more than 12,000 children have been trained in the various Homes, and that 11,000 have been placed out in situations equipped for earning a livelihood, while 1,000 are now under training. Lord Jersey (the President) has shown that though separated by many miles from this country, he still keeps the Institution in remembrance. He provided before leaving for Australia that the usual arrangements should be made for giving the children of the Homes a holiday at Osterley Park. He, as well as the Committee of Management, will no doubt have felt gratified to find that boys who had been sent to sea had not forgotten an invitation he gave all who might be cruising or travelling in the Antipodes to come and see him at Government House. Though other institutions have sprung up to carry out the same objects as this, which has now been doing such good work for nearly fifty years, we cannot help thinking that the machinery of the old Society would have sufficed to do all the necessary work if properly supported. The multiplication of societies on similar lines cannot but lead to wasted energy and unnecessary expenditure.

Mr. John S. Gilliat, M.P., who presided at the recent anniversary festival of **THE ROYAL ASYLUM OF ST. ANNE'S SOCIETY**, did but re-echo the feelings of the majority of people when he said that those who had seen better days and whose associations had fitted them for a higher sphere of work are eminently worthy of generous sympathy and support in endeavouring to raise themselves again to the position from which they have fallen. The sufferings of those who have known better things are infinitely greater and harder to bear than those of people who have never known the so-called pleasures of life, but have been brought up from their very infancy amidst squalor and poverty. It cannot be made known too widely that those who have been in prosperity, and have been overtaken by some calamity over which they had no control, are the very ones who hang back and are most reluctant to let the world know that they are in distress. The saddest troubles in the world are not those which are paraded before our eyes in the public thoroughfares, but those which have to be sought out, and of people who, from a laudable sense of pride, shrink from disclosing their sorrows. The work of a society which helps such as these is worthy of the most generous support and sympathy from the well-to-do. The work done by the schools of the Society at Redhill is truly important, and it is to be regretted that out of the 130 children waiting last month for admission to the schools, only 25 per cent. could be taken, the remainder having to be refused admission owing to want of funds. There is room in the building for twenty more beds, and it is only required that the charitably disposed public should come forward in order that th y may be filled up. The firm of which Mr. Gilliat is a member have shown their practical sympathy with the objects of the institution by contributing a donation of one hundred guineas.

### Scraps and Gleanings.

The tenth anniversary service in connection with the Sarah Nicol Cottage Hospital at Llandudno was very largely attended.

Lady Alexander Gordon Lennox has given two concerts in aid of the St. Elizabeth's Hospital, which were highly successful.

The managers of the Sick Children's Hospital at Edinburgh have definitely decided to rebuild the hospital on the present site.

Mr. Dowell has resigned the post of Treasurer to the West End Hospital, and Mr. A. Bedborough has been appointed in his place.

The Hull Infirmary Samaritan Fund is urgently in need of subscriptions, which may be sent to Miss Cox, the Lady Superintendent.

A British Association of Cooks, Confectioners, and Pastrycooks has been formed for the advancement of the art throughout the country.

The plans of Mr. J. W. Donald, of South Shields, have been selected for the new buildings in connection with the Sunderland Eye Infirmary.

The Duke of Westminster will visit the National Hospital for the Paralysed and Epileptic on the 18th inst. to inaugurate the new surgical wing.

Madame Adelina Patti will give a grand morning concert at the Albert Hall on the 27th of August in aid of the funds of the Swansea Hospital.

At the 74th annual meeting of the subscribers to the Swansea Hospital it was stated that 1,120 in-patients and 2,500 out-patients had been treated during the year.

Hospital Saturday Fund collections will be made at the end of the present week. As many as 2,300 lady collectors will take up positions in the metropolitan thoroughfares.

The Bishop of Durham, speaking on the subject of convalescent homes, stated that he considered that there was no form of philanthropy to be more cordially commended.

The half-yearly meeting of the Devonshire Hospital was held on the 4th inst., Dr. Robertson, M.D., in the chair. The results shown in the medical report were highly satisfactory.

The third annual report of the Hull Orthopædic Hospital shows that 372 patients have been treated. The hospital has been removed to new and more sanitary premises in Wright Street.

It is announced in Chicago that a British Syndicate has been formed for the purpose of utilising the great lakes in order to establish a continuous water communication between Chicago and Great Britain.

According to a report issued this week, the total number of persons killed on railways during the first three months of the present year was 322, and 3,101 were injured in the same period; of these 22 were suicides.

The influenza has proved itself a most debilitating illness during the present spring and summer. Although it is months since Mr. John Morley was first attacked, he is only now recovered sufficiently to look forward to an early resumption of public life.

The report of the Royston Cottage Hospital shows a deficiency of £40 on the year's working. The Committee have had to borrow the money from the Building Fund, but we hope so worthy a charity will receive more adequate support during the current year.

At a demonstration of friendly societies, which had already collected £3,000 for various charities, for the benefit of the Hartlepool Hospital, Mr. Furness, M.P., stated that he would himself supply any deficiency should the sum collected on this occasion fall short of the usual average of £50.

The Marquis of Carmarthen, M.P., presided at the annual meeting of the London Skin Hospital at the Whitehall Rooms, Hotel Métropole. The fourth annual report showed theat during the past year there were admitted to the institution 1,189 new out-patients, and the attendances numbered 7,601.

The Earl of Wemyss and March presided on the 13th inst. at a banquet to Major Wm. Vaughan Morgan, in recognition of his services as Chairman of the London Homœopathic Hospital, and presented him with the sum of £4,000 to complete the total of £30,000 required for rebuilding the Hospital.

The annual report of the Great Yarmouth Hospital shows that 379 in-patients and 3,505 out-patients have been treated during the year. The Hospital Saturday collection was the smallest ever held, and it is satisfactory to note that there is a balance of £60 in favour of the hospital on the year's account.

The Ancoats Hospital, Manchester, which had only forty beds in 1886, now contains 76 beds, the pressure on which is so great that an appeal has been issued with the view of adding a further 27 beds to the hospital. There has been an increase of about 30 per cent. in the number of patients during the last three years.

Mr. Webster states that the workmen of London, exclusive of payments to provident dispensaries, contribute £87,793 to the London hospitals during the year. As this figure includes £48,528 from patients' payments, and about £10,000 from boxes, it will be seen that the statement cannot be accepted as accurate or convincing.

The Convent of St. Victor, near Montreal, which is used as an asylum for deaf mutes, was, last week, burned almost to the ground. The inmates numbered 300, and a large number of them had very narrow escapes, the stairs having quickly caught fire and become impassable. Sister Margaret prevented a terrible loss of life. She made a rope by tying together a number of sheets from the beds, and thus lowered 140 of the inmates safely to the ground through the windows.

At a convention of lady abstainers at Melbourne, comprising delegates from all the Australian colonies, the sensational paper was read by Fraulien Lepper, who made a determined assault on a long-standing feminine institution. "In my opinion," she said, "next to alcohol, the greatest master of the human will and destroyer of vitality is tea. Theine is one of the important elements in tea. If theine is given in sufficient doses to animals it first paralyses them, after which they become convulsed and die."

### THE STUDENT OF MEDICINE.

[All communications for this Supplement should be addressed to THE EDITOR of THE HOSPITAL, at 140, Strand, with the words, "Students' Column," written in the left hand top corner of the envelope.]

### EDITORIAL NOTES.

GIRTON COLLEGE.—This well-known Ladies' College at Cambridge has just received a valuable addition to its recommendations in the form of a prize founded by Mr. Claude G. Montefiore, in memory of his late wife, a certificated student in honours of the College. The prize, which is called the Therese Montefiore Memorial Prize, consists of the annual interest on £1,700. It is to be given yearly, on the recommendation of the mistress and vice-mistress of the college, to a student of not less than three or more than four years' standing who shall have obtained a first class in one of the triposes of the University of Cambridge, and who at the time of election shall be intending to follow either the teaching or the medical profession, or to pursue some specific literary or scientific work.

SOCIETY OF APOTHECARIES OF LONDON.—The results of the examination in arts qualifying for registration as medical students, held in the hall of the society on June 5th and 6th, has just been published. There were 171 candidates, and from the Pass List it appears that one was placed in the first class and 24 in the second class, and 111 were certified as having passed in some subjects but not in all. The next examination will be held on September 4th and 5th, and again on December 4th and 5th.

### ON GERMAN STUDENTS.

*The substance of a paper read before the St. Mary's Hospital Medical Society by* W. OVEREND, *B.A. Oxon.*

[It was with considerable hesitation that I prepared the following paper, seeing that I can only draw upon my own experiences, and these have been limited to the Universities of Strasburg and Heidelberg. The title of the paper, therefore, is somewhat of a misnomer.

The German students at the age of 17 or 18 pass their matriculation from the Gymnasium or High School, and then proceed to the University.

This paper will begin with the teaching of the intermediate subjects—Anatomy and Physiology. In anatomy the German methods are somewhat different from ours. In the first place, histology is more definitely associated with the teaching of anatomy than in England. The dissecting room at Strasburg was open from nine to six daily, commencing with a lecture at nine, the first assistant being present in the mornings and the two professors, Schwalbe and Joessel, every afternoon. As regards the parts for dissection, the bodies are first injected and then cut up as follows: The whole body is bisected longitudinally, the viscera are removed, and each half is again divided into two parts, one including the head, neck, upper extremity, chest wall, and back, and the other the pelvis and lower extremity. The arteries and nerves are dissected on different parts. This is for the second year's men. The first year's men only dissect muscles on the intact body, and the viscera after they have been removed. After having dissected the vessels, nerves, and muscles on the injected body, the student spends the rest of his time in the dissection of special regions on the uninjected body, such, for instance, as the axilla, the popliteal space, the triangles of neck, the perinæum, the bend of the elbow, &c. Parts are exceedingly plentiful, and provided gratuitously, the fee for a course of six months' dissection being only 35s. When a student has finished his day's work the parts are placed for the night in large tanks containing spirits of wine. The two professors are present during the whole afternoon, and each worker receives a large amount of individual attention from them.

In *Physiology* the teaching, on the whole, does not seem to be so good as in England, although many of the foreign professors are of world-wide renown, such as Goltz, in Strasburg, and Kühne, in Heidelberg. There is a lecture daily, and Kühne usually gave a demonstration every Saturday on the subject of the week. These demonstrations were exceedingly good and untrammelled by legislative restraints. For instance, a demonstration on blood-pressure was given on a large dog under curare, a manometer being placed in the crural artery, and the movements registered on a travelling surface. By stopping artificial respiration, he demonstrated the effect of various phases of asphyxia on the blood pressure curve with the sweeping undulations of Traube-Hering. Then artificial respiration was resumed,

### The Hospital Advertiser.

### MEDICAL APPOINTMENTS.

### THE HOSPITAL FOR SICK CHILDREN,

8th July, 1891.

### THE HOSPITAL FOR SICK CHILDREN,

THE STUDENT OF MEDICINE—(*continued*).

and the effect of stimulation of the vagus was demonstrated. The students themselves do very little practical work, and their knowledge of histology is exceedingly limited. The courses in operative surgery are held in the dissecting-room during the winter semester three times weekly, from six to seven in the evenings. At the end of the second year the student enters for his intermediate examination, which is known as the Vorprufung or Physicum, and includes anatomy and physiology, with biology, chemistry, and physics. This examination is purely *vivâ voce*, there being a viva in each subject, lasting about twenty minutes. It is by no means difficult, provided the student has a proper appreciation of general principles. Should a student fail in one or more subjects he takes them up again after the lapse of a few weeks. From personal acquaintance with several of the students I know, as a fact, that the standard is very low. The student must pay the fees for a certain number of lectures, but whether he attends or not is purely optional. There is no signing up as in this country.

After the Physicum is passed, the student commences to attend the clinical lectures. As a rule the routine is as follows: The gynæcological clinic, from 8 to 9; then the surgical and medical, from 9.15 to 12 (about 75 minutes each); and the eye clinic, from 12 to 1. In the afternoon, pathology lectures from 2 to 3, and post-mortem demonstrations twice a-week from 3 to 4: after which follow the ordinary lectures on medicine and surgery, which finish about 6.45. There is much to be said in favour of these clinical lectures, combining, as they do, theory with practice. In the clinics one student is selected in turn to examine cases before the class, or he first goes into the ward to examine the case, and the patient is subsequently brought into the lecture-room. If the patient is too ill to be moved, the whole of the students proceed into the wards, where seats are provided for them. When this happens it need scarcely be said that the majority see very little. In Strasburg there was no room for the students in the wards, so the cases were invariably introduced into the theatre. On occasions, though I must confess these were rare, the patient was in much too grave and serious a condition to be subjected to rude shocks and draughty corridors. Possibly he was brought in on the off-chance of a post-mortem. It is, however, unnecessary for me to enter into the question of the treatment of the patient, humane or otherwise; in fact, the less said the better.

Professor Erb is the director of the medical clinic at Heidelberg. He is a nerve specialist, but he proved himself none the less *au courant* in every department of medicine, and especially conversant with the recent additions to its literature. As an illustration of one of his lectures, we may give the following memoranda: First case: *Cystitis.* Second case: *Chronic eczema.* Third case: *Case of tetany*, in which he demonstrated the increased excitability of the facial nerves to the anode of the galvanic current, and the phenomenon of evoking the characteristic position of the hand when the brachial artery is compressed. Fourth case: *Paralysis agitans*, which he thoroughly investigated, in this case demonstrating the influence of subcutaneous injection of hydrobromate of hyoscyamine, which stopped the tremours in about a quarter of an hour, an effect lasting several hours. Fifth case was one of *diabetes mellitus*, which was fully gone into. He demonstrated the presence of acetone in the urine, and discussed the pathology of the disease. Sometimes he would take up some special subject, as phthisis or typhoid, and among other cases would demonstrate one of these every morning for about nine consecutive days, after which he would give one lecture on the whole, comparing, summarising, and entering into the question of diagnosis, complications, and treatment.

The practice of asking each student in his turn to examine a case in the wards, which on his return must be carefully described, with the history, diagnosis, and treatment, is excellent. The student is obliged to speak at least five or six minutes, and often speaks fluently and well. One cannot help, indeed, noticing their ready flow of language. To some extent this may be accounted for, not by racial differences, but by practice and necessity. The student takes out a certain number of lectures, although his attendance at the same is not recorded. During the lecture their attendance and conduct is irreproachable, and note-taking is the rule. We might here point to the utter lack of humour exhibited by the students. Whether it is he has such an exalted opinion of the dignity of his own profession, or is too conceited for frivolity, or possibly gets too little muscular exercise, since active sports, football, cricket, &c., are looked upon more or less with horror, or seeing that the medical profession is considerably more overcrowded than with us, and this emphasises the Darwinian axiom of the survival of the fittest, certainly it is a fact, that the most ridiculous occurrences during the lectures awaken none of their

THE STUDENT OF MEDICINE—(*continued*).

visible faculties. I remember Professor Becker questioning a student on an eye case—the student described the signs most carefully, but had no idea as to the diagnosis. It was a glass eye, and when Becker removed it, not the faintest shadow of a smile was perceptible, either in the professor or any of the students. If there is any disturbance among the students, it is either to get at a microscopic specimen, or to get into the theatre first in order to secure a place.

(*To be continued.*)

### NOTES FROM THE SCHOOLS.

### Edinburgh University.

The E.U.A.C. twenty-fifth annual sports were held at the University Ground, Corstorphine, on July 4th. Results:—Throwing the cricket ball (confined): 1, H. M. Crosby, 98 yards 1 foot; 2, T. A. Chalmers, 92 yards. 100 yards flat race (confined): 1, R. Wilson; 2, F. Atkinson. 10 3-5 sec. 1,000 yards handicap (confined): 1, D. S. Duncan scratch; 2, G. W. Pollard, 30 yards; 3, R. T. Mitchell, 33 yards. 2 min. 27 1-5 sec. Throwing the hammer (confined): 1, T. M. Donovan, 93 ft. 8 in.; 2, M. M. Innes. Place kick at goal (confined): 1, A. J. Nevitt. High Jump (confined): 1, R. Williams, 5 ft. 4 in.; 2, C. W. Branch. 220 yards flat race handicap (open): Final heat—1, H. W. Price, S.C.A.C., 20 yards; 2, G. Munro, E.U.A.C., 18 yards; 3, A. Steel, E.U.A.C., 9 yards. 23 sec. Half-mile flat race (confined): 1, D. S. Duncan; 2, S. B. Figgis. 2 min. 29 4-5 sec. One mile safety handicap: 1, J. Anderson, Wav. Roads Club, 25 yards; 2, A. Alexander, E.T.C.C., 110 yards; 3, R. Carmichael, S.C.C.C., 85 yards. Time, 3 min. 20 3-5 sec. 120 yards handicap (confined): Final heat—1, A. Steel, 4 yards; 2, J. C. Shelmerdine, 9 yards; 3, G. Munro, 9 yards. Time, 12 3-5 sec. Three mile team race for University Cycling Challenge Cup: 1, Edinburgh Southern C.C. team—J. Davie, A. Duff, W. J. Muir. The other teams were Edinburgh Eastern and Edinburgh University. The University have lost the cup five years and held it six. 120 yards hurdle race (confined): 1, T. M. Donovan; 2, H. W. G. Lander. 17 sec. One mile race (confined): 1, D. S. Duncan; 2, R. T. Michell. 4 min. 56 1-5 sec. Putting the weight (confined): 1, M. M. Innes, 31 ft. 4 in.; 2, T. A. Chalmers. Long jump (confined): 1, T. M. Donovan, 20 ft. 8 in.; 2, R. Williams, 20 ft. 4 in. 880 yards handicap: 1, J. M. Gow, S.C.A.C., 45; 2, D. M'Lagan, E.H., 30; 3, A. W. Forrest, E.U.A.C. and E.H., 30. 2 min. 8 1-5 sec. Two miles bicycle handicap (confined): 1, R. Sanson, scratch; 2, A. Cameron, 80 yards. 440 flat race (confined): 1, T. M. Donovan; 2, H. W. G. Lander. 54 1-5 sec. One mile handicap (open): 1, G. W. Pollard, E.U.A.C., 80 yards; 2, A. Reid, E.N.H., 100 yards. 3, D. M'Lagan, E.H., 75 yards, 4 min. 43 1-5 sec. Three miles safety handicap: 1, A. Duff, E.S.C.C., 250 yards; 2, J. Anderson, Wav.Rds.C.C., 50 yards; 3, A. Alexander, E.T.C.C., 250 yards. 10 min. 1 3-5 sec. 440 yards flat race handicap (open): 1, D. L. Anderson; 2, A. Grierson; 3, H. W. Price, S.C.A.C. Time, 53 sec. The wind was very strong and bothered the competitors, especially in the cycle races. There was no restrictions in the cycle events as to tyres, and every one rode pneumatics. T. M. Donovan won the Australasian Cup and championship of the E.U.A.C. for the second year, beating D. S. Duncan by 23 points to 15. At the close the prizes were presented by Mrs. Annandale.

### APPOINTMENTS.

At Guy's Hospital, J. H. Badcock, M.R.C.S., L.R.C.P., L.D.S., A. E. Baker, M.R.C.S., L.R.C.P., L.D.S., J. O. Butcher, L.D.S., and P. Traer Harris, M.R.C.S., L.S.A., L.D.S., have been appointed assistant dental surgeons. H. V. Hickman has been reappointed ophthalmic assistant, and Montague F. Hopson, L.D.S.Eng., has been appointed dental house surgeon. At the Hospital for Diseases of the Chest, Brompton, Charles J. Stanley, M.B.Durh., M.R.C.S., L.R.C.P.Lond., has been appointed house physician. At the Alexandra Hospital for Diseases of the Hip, Frances May Dickinson, M.B., has been appointed anæsthetist. At the Belmont Road Infirmary, Liverpool, J. L. Henstock, L.R.C.P., M.R.C.S., has been appointed assistant medical officer. At the County Hospital, Durham, Selby W. Plummer, M.B., B.S.Dunelm, has been appointed house surgeon. At the Hants County Asylum, Knowle, Fareham, Francis O. Simpson, L.R.C.P., M.R.C.S., has been appointed third assistant medical officer. At the Ingham Infirmary and South Shields and Westoe Dispensary, W. C. Haswell, M.B., B.S.Durh., has been appointed junior house surgeon. At the Liverpool Northern Hospital, C. Brooks, L.R.C.P., M.R.C.S., has been appointed house physician;

### THE STUDENT OF MEDICINE—(*continued*).

and S. J. Palmer, M.B.Durh., M.R.C.S.Eng., assistant house surgeon. At the Royal South London Ophthalmic Hospital, William Job Collins, M.S., F.R C.S., has been appointed honorary assistant surgeon. William Henry Russell Forsbrook, M.B.Lond., M.R.C.S.Eng., L.S.A., has been appointed consulting medical officer in London to the Government of the Cape of Good Hope. At the Home and Infirmary for Sick Children and South London Dispensary for Women, Sydenham, F. J. Lorimer Hart, M.B. and C.M.Edin., has been appointed honorary assistant surgeon.

### VACANCIES.

The following vacancies are announced this week, the amount of salary in each case being given after the name of the institution:

#### Resident Medical Officers.

Applecross Parochial Board, Medical Officer—Salary £95.
Birmingham Gen ral Dispensary, Surgeon—Salary £150, &c.
Derbyshire Royal Infirmary, Assistant House Surgeon—Salary £10, &c.
General Hospital, Birmingham, three Assistant House Surgeons.
Ingham Infirmary and South Shields and Westoe Dispensary, Senior House Surgeon—Salary £60, &c.
Kensington Dispensary, Resident Medical Officer—Salary £125.
Lancashire County Asylum, Rainhill, near Liverpool, Assistant Medical Officer to act as Pathologist—Salary £200, &c.
Lunatic Hospital, Nottingham, Assist. Med. Officer—Salary £100, &c.
Metropolitan Asylums Board, Clinical Assistant for the South Eastern Fever Hospital, New Cross Road, S.E.
Norfolk and Norwich Hospital, Norwich, Assistant to House Surgeon.
Royal Albert Hospital, Devonport, Assistant House Surgeon.
Royal Albert Edward Infirmary and Dispensary, Wigan, Senior House Surgeon—Salary £100.
Sheffield General Infirmary, Assistant House Surgeon—Salary £80, &c. House Surgeon—Salary £120, &c.
Western General Dispensary, Marylebone Road, N.W., Junior House Surgeon—Salary £50, &c.
Bradford Infirmary and Dispensary, Dispensary Surgeon—£100, &c.

#### Non-Resident Medical Officers.

Grosvenor Hospital for Women and Children, Vincent Square, Westminster, S.W., Assistant Physician.
Hampstead Provident Dispensary, New End, N.W., Medical Officer.
Hospital for Sick Children, Great Ormond Street, Assistant Physician.
Middlesex Hospital, W., Second Chloroformist.
Sheffield School of Medicine, Tutor to take charge of Dissecting Room and Hold Classes in Anatomy and Physiology—Salary £100.
University of Durham College of Medicine, Newcastle-on-Tyne, Demonstrator of Anatomy—Salary £100.

### Prize Distribution at Guy's Hospital.

The annual distribution of medals and prizes to the successful students of Guy's Hospital Medical School took place on Wednesday, the 8th inst. The weather turned out most unfavourable for the arrangements which had been made for the reception of the visitors, a platform having been erected in the quadrangle opposite the colonnade, which was elegantly decorated with flowers and banners. Shortly before the appointed hour several students and their friends, including a large number of ladies collected, but as rain began to fall it was thought advisable for the proceedings to take place in the lecture theatre. As, however, the rain soon left off, Mr. J. A. Shaw-Stewart presided over the meeting in the quadrangle. But as soon as the proceedings were opened rain again commenced to fall, and this time in real earnest, and a heavy thunderstorm broke overhead. At times the thunder and noise from the rain was so loud as to drown the voices of the speakers. Dr. Perry, the Dean of the Medical School, read his report, in which he referred to the prosperity of the school, as shown by the increase in the number of entries during the past year, the total for the year being 164 as compared to 144 in the previous one. With reference to the Dental School, Dr. Perry spoke of its vigorous growth, and said that, whereas in October, 1889, it started with six students, they now numbered over 30. Patients had also increased, and new accommodation was about to be provided for them. During the ensuing winter session there would be a course of instruction in public health for the diploma now given by qualifying bodies upon the recommendation of the General Medical Council. Special chemistry classes had also been arranged for. Mr. J. A. Shaw-Stewart then proceeded to distribute the medals and prizes, amid hearty applause. They were awarded as follows: The Treasurer's gold medal for clinical medicine, Mr. John Henry Bryant, Ilminster, who also took the Treasurer's gold medal for clinical surgery and the Beaney prize for pathology. The Gurney-Hoare prize for clinical study, Mr. Arthur Stanley Wohlmann, Hertford; the Golding-Bird prize and gold medal, Mr. Alfred Theodore Rake, Fordingbridge, and the Michael Harris prize for anatomy, Mr. Guy Eugene Manning, Hammersmith. The open scholarships in arts prize, value 100 guineas, was taken by Mr. Henry Percival Ferraby, of Bedford; whilst Mr. Francis James Steward, of Old Charlton, carried off the open scholarships in science, prize, value 1[illegible]5 guineas. The first year's students' prize (1890), of £50, was awarded to Mr. Arthur Henry Leete, of Wellingborough. A vote of thanks to Mr. Shaw-Stewart, proposed by Mr. E. H. Lushington (Treasurer of the hospital) concluded the proceedings. The wards, museums, school buildings, college, and chapel, were then inspected by the visitors, and the band of the M Division of Police, by permission of Mr. Superintendent Denis Neylan, performed a selection of music in the quadrangle.

